# The Role of Dectin-1-Mediated M1 Macrophage Polarization in Cerebral Ischemia-Reperfusion Injury

**DOI:** 10.1155/2021/6697271

**Published:** 2021-05-21

**Authors:** Chen Zongyun, Lin Bixia, Ding Fadian, Hong Xiaoping, Chen Hongbin, Deng Yu, Liu Qicai, Ye Xiaoyi, Zeng Kai

**Affiliations:** ^1^Department of Laboratory Medicine, Mindong Hospital Affiliated to Fujian Medical University, Fuan 350000, Fujian Province, China; ^2^Department of Pharmacy, The First Affiliated Hospital of Fujian Medical University, Fuzhou, Fujian Province, China; ^3^Department of Surgery, First Affiliated Hospital, Fujian Medical University, Fuzhou 350000, Fujian Province, China; ^4^Department of Ophthalmology, First Affiliated Hospital, Fujian Medical University, Fuzhou 350000, Fujian Province, China; ^5^Nano Medical Technology Research Institute, Higher Educational Key Laboratory for Nano Biomedical Technology of Fujian Province, School of Pharmacy, Fujian Medical University, Fuzhou 350000, Fujian Province, China; ^6^Center for Reproductive Medicine, First Affiliated Hospital, Fujian Medical University, Fuzhou 350000, Fujian Province, China; ^7^Department of Nephrology Medicine, Mindong Hospital Affiliated to Fujian Medical University, Fuan 355000, Fujian Province, China; ^8^Department of Anesthesia, The First Affiliated Hospital of Fujian Medical University, Fuzhou 350000, Fujian Province, China

## Abstract

**Introduction:**

The advances in cerebral ischemia treatment have resulted in a larger proportion of patients get the benefits of rebuilding blood flow to the brain. Then, ischemia-reperfusion injury has emerged as a new essential problem. Dectin-1 plays an important role in cerebral ischemia-reperfusion injury by regulating the function of immune cells.

**Methods:**

C57BL/6 was blindly divided into four groups including the sham-operated group and the three different kinds of middle cerebral artery occlusion (MCAO) groups (after 6 hours, 12 hours, and 24 hours after plug removal). The protein expression levels of dectin-1, proapoptosis molecule, and antiapoptosis molecule were measured by using western blot analysis. The brain tissue was analyzed by flow cytometry to detect the M1 macrophage levels.

**Results:**

The correlation analysis of dectin-1 and infarct areas showed that there was an obviously positive correlation in between them (*R* = 0.9603). Dectin-1, cleaved caspase-3, and Bax increased, while antiapoptosis molecule, Bcl-2, decreased at three appropriate time points (after 6 hours, 12 hours, and 24 hours). The level of M1 macrophages in the experimental group increased after ischemia-reperfusion injury compared to the control group.

**Conclusions:**

The high expression level of dectin-1 may affect M1 macrophage polarization and brain cell apoptosis in cerebral ischemia-reperfusion injury.

## 1. Introduction

Stroke was one of the leading causes of disability and the second highest cause of death globally, with certain obviously increasing prevalence in some areas [1], while ischemic stroke is accounted for approximately 72.5% of strokes overall [2]. Of note, stroke was the main cause of neurological function loss and neuronal death during the process of cerebral ischemic injury [3]. Brain reperfusion injury following ischemic stroke was defined as damage of brain tissue suffered from insufficient blood supply which dramatically reduced the benefits of new establishing blood flow for acute ischemic stroke [4].

Inflammation was a crucial factor associated with the progression and prognosis after cerebral ischemia-reperfusion injury. Previous studies had shown that the abnormal activation of residential microglia which were derived from glial cells and macrophages which were derived from circulating monocytes contributed to the acute cerebral ischemic injury [5]. The peripheral immune-related cells include T lymphocytes, B lymphocytes, and neutrophils, which also aggravated neutrophil function damage by releasing proinflammatory cytokines in the early stage of stroke [6, 7]. Bone marrow-derived macrophages accelerated inflammation and injury in the brain infarcted areas after ischemia-reperfusion, which increased the expression levels of angiopoietin-like protein (ANGPTL) 2 [8].

Dectin-1 is one of the members of the C-type lectin receptor (CLR) family, which involved in numerous pathophysiological processes including neuroinflammation [9], macrophage polarization, and neutrophil infiltration [10]. Based on several previous research studies, dectin-1 was mainly expressed on dendritic cells, macrophages, and neutrophils [11], which was involved in ischemia-reperfusion injury by participating in the regulation of macrophage polarization and neutrophil infiltration [10]. Up to date, there are still very limited studies in this field, so further exploration is needed to explore the potential role of dectin-1 involved during the development of cerebral ischemia-reperfusion injury. In this study, we investigated the expression levels of dectin-1 in the brain and explored the functional mechanism of dectin-1 after cerebral ischemia-reperfusion injury.

## 2. Materials and Methods

### 2.1. Animal

Male C57BL/6 mice (6–8 weeks old, 18–22 g) were directly purchased from the Animal Experimental Center of Three Gorges University. All mice were maintained in a specific-pathogen-free (SPF) environment in 3 cages (8 rats/cage in total) with 4 groups divided individually (2 rats/group), and the environment was 22–25°C with adequate food and water. All animal-related studies complied with the National Institutes of Health guidelines for the care and use of laboratory animals. Animal-related studies were carried out by Ding Fadian in the animal laboratory center of Wuhan Barfil Biotechnology Co., Ltd. Animal-related studies were approved by the Ethics Committee of Mindong Hospital affiliated to Fujian Medical University.

### 2.2. Model of Ischemic Stroke

The rats were of the same range of age at the start of the experiment. C57BL/6 were anesthetized with 5% isoflurane, and the anesthetization condition was maintained with 1.5% isoflurane. The mice were fixed on the operating plate in supine positions, and the skin on the neck was disinfected. The submandibular gland could be observed by removing the skin along the middle of the neck in the process of blunt separation. The gland was gently pushed to one side to separate to the tracheal anterior muscle and then separated down along the right sternocleidomastoid tendon. After seeing the carotid sheath, the skin muscle was fixed with a retractor. At this point, the common carotid artery was seen. The common carotid artery away from the bifurcation was ligated, and a slip knot was left at the common carotid artery near the bifurcation, and the external carotid artery near the bifurcation was ligated. A small incision in the common carotid artery was made by microscopic shear. We picked up the MCAO line tied through the blood vessels and gently lifted the silk thread of the common carotid arteries' ligation. Then, we loosened the vascular clamp when the line tied enters the internal carotid artery, and we continued to push the line tied until the marker reaches the location of the vessel bifurcation. Tighten the slipknot. After 60 minutes of ischemia, the plug was removed, and the live mice were ligated to death. The muscle and skin were sutured. Samples were taken 6, 12, and 24 hours later. The success of the model was determined by Zea-Longa's method [12]. The neurological function of the rats was scored on a scale of 0–4. The behavior scores were evaluated 24 h after operation. The animals with scores ranging from 1 to 3 were considered successful, and animals with scores of 0, 4 and unstable vital signs were rejected.

### 2.3. Triphenyl-2,3,5-tetrazoliumchloride (TTC)

The sections of the brain of the mice were put in 2% triphenyl-2,3,5-tetrazoliumchloride (TTC) at a 37°C temperature box for 30 minutes. Each slice was fully soaked in TTC and washed with PBS. The result of TTC was photographed with a digital camera blindly. The white areas of sections (infarction area) and red sections (noninfarcted area) were measured using ImageJ, and the total infarct area is the sum of the infarcts (infarction area/(noninfarcted area + infarction area)).

### 2.4. Western Blot

The brain tissues were cut, grinded, and lysed with RIPA lysis and extraction buffer (Cat #P0013B, Beyotime), and protease and phosphatase inhibitor were added (Cat #P105539, Aladdin). Proteins (30 *μ*g) were separated by SDS-PAGE (5% concentrated gel and 12% separating gel) and transferred to PVDF membranes (Cat #ISEQ15150 and Cat #IPVH00010, Millipore). 5% nonfat milk was used to block, and the PVDF membranes were incubated with targeted primary antibodies and secondary antibodies. At last, the protein bands were visualized with an ECL detection solution (Thermo). The primary antibodies were as follows: rabbit polyclonal antibody dectin-1 (Cat #Ab140039, 1 : 1000; Abcam), mouse monoclonal antibody Bcl-2 (Cat #Ma1-12246, 1 : 1000, Thermo), mouse monoclonal antibody Bax (Cat #Sc-7480, 1 : 1000, Santa), rabbit polyclonal antibody caspase-3 (Cat #9662, 1 : 1000, Cst), and anti-GAPDH (Cat #AB-P-R 001, 1 : 5000, Hangzhou Xianzhi Biology Co., Ltd). We quantified the intensity of the protein bands with ImageJ software, while all the target proteins normalized to GAPDH.

### 2.5. Flow Cytometry

The brain tissue was cut into pieces with scissors and digested with 0.25% trypsin-EDTA complex digestive solution at 37°C for 30 min. Serum terminated the digestion, and the falcon filtered the cells. After centrifugation, the liquid was discarded. PBS-resuspended cells with 1 mL 0.5% bovine serum albumin (BSA) per flow tube were centrifuged at 1000 rpm for 3 min. The supernatant was discarded, and PBS was added to resuspended cells to be measured. Antibodies CD11b (eBioscience™) and F4/80 (eBioscience™) were added and incubated at 4°C in dark for 30 min, after which the cells were measured using the flow cytometer (Beckman Coulter). FSC and SSC were set in the first experiment based on the cell size of macrophages. In the following experiments, these were not changed.

### 2.6. Statistical Analyses

Statistical analyses were performed using GraphPad Prism 5.0 (GraphPad Prism Software Inc., San Diego, CA, USA) and SPSS 15.0 for Windows (SPSS Inc., Chicago, IL, USA). Continuous variables were expressed as median (interquartile range, IQR) and calculated by Mann–Whitney test. Categorical variables were expressed as percentage and compared by test between groups. *P* < 0.05 was considered statistically significant.

## 3. Results

### 3.1. The Expression Level of Dectin-1 in the Brain Tissue Increased after Cerebral Ischemia-Reperfusion Injury

**Figure 1:**
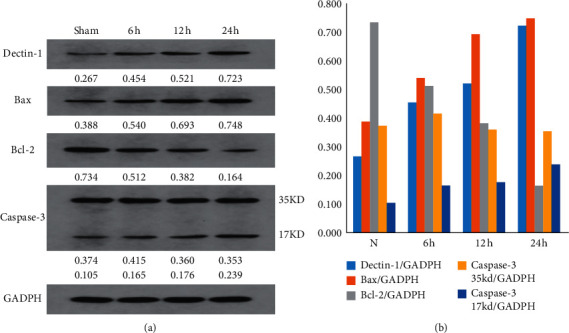
The expression level of dectin-1 and infarct area. (a, b) The sham-operated group and the middle cerebral artery occlusion (MCAO) group (after 6 hours, 12 hours, and 24 hours).

**Figure 2:**
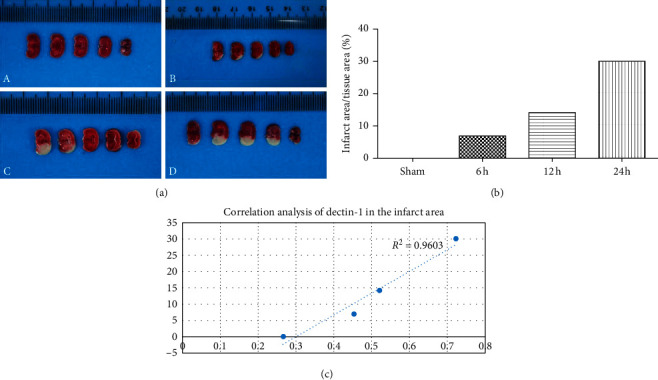
Flow cytometry analysis of macrophage in the model of cerebral ischemia-reperfusion injury. (a) It is labeled with CD11b-FITC and F4/80-PE showing uniform and complete labeling of M1-polarized macrophages: (A) sham-operated group; (B) MACO 6 h; (C) MACO 12 h; (D) MACO 24 h (b) Ratio of infarct size. (c) Positive correlation between Dectin-1 and infarct size.

To explore the potential mechanism of dectin-1 in the cerebral ischemia-reperfusion injury, we investigated the expression levels of dectin-1 in the infracted brain tissue at three appropriate time points after the ischemia-reperfusion injury. Protein expression levels of dectin-1 were shown with an increasing trend at 6 hours, 12 hours, and 24 hours after ischemia-reperfusion. Meanwhile, the result of TTC showed that the rate of ischemic area/tissue was dramatically increased (see Figure 1(a)). After ischemia-reperfusion, the ratio of the protein level of dectin-1/GADPH was 0.454, 0.521, and 0723 at 6 hours, 12 hours, and 24 hours, respectively, while in the sham-operated group, it was 0.267. The ratio of the ischemic area/tissue area was 6.92%, 14.11%, and 30% at 6 hours, 12 hours, and 24 hours, respectively, while in the sham-operated group, it was observed without any respective increase (see Figure 1(b)). The correlation analysis of dectin-1 in the infarct area showed that it was an obviously positive correlation (*R* = 0.9603) (Figure 2(c)). The expression level of dectin-1 was significantly correlated with cerebral infarction after cerebral ischemia-reperfusion injury.

### 3.2. Dectin-1 Was Involved in Cerebral Infarction after Cerebral Ischemia-Reperfusion Injury by Mediating Apoptosis

**Figure 3:**
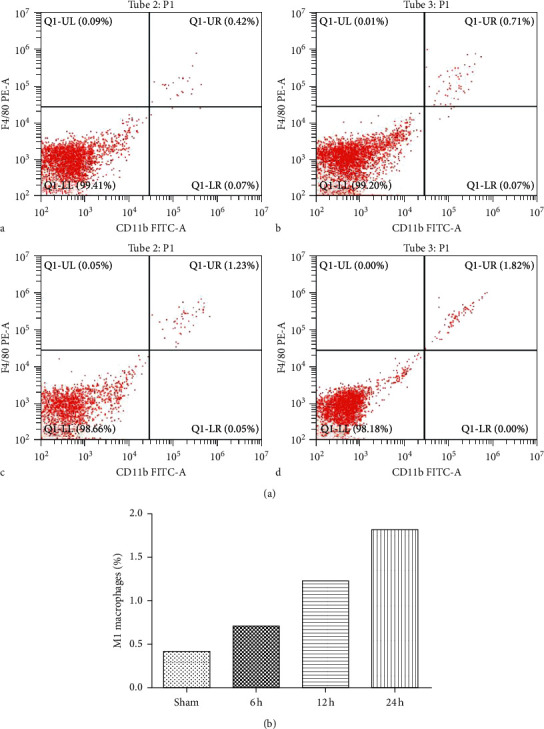
Dectin-1 was involved in cerebral infarction after cerebral ischemia-reperfusion injury by mediating apoptosis. (a) The sham-operated group and the middle cerebral artery occlusion (MCAO) group (after 6 hours, 12 hours, and 24 hours). Dectin-1, Bax, and cleaved caspase-3 increased, and antiapoptosis molecule, Bcl-2, decreased at three appropriate time points compared to the sham-operated group. (b) The quantitative analysis of western blot.

Based on previous studies, the interference of dectin-1 by genetic ablation or antibody blockade led to a considerable improvement in cardiac function, accompanied by an increase of apoptosis-related protein [10]. To further investigate the underlying molecular mechanisms in the cerebral infarction, we measured the protein levels of dectin-1, proapoptosis molecule, and antiapoptosis molecule. After the ischemia-reperfusion injury, representative western blot analyses and summary data showed the protein levels of proapoptosis molecules Bax and cleaved caspase-3 decreased, while antiapoptosis molecule Bcl-2 increased at three appropriate time points (6 hours, 12 hours, and 24 hours), in comparison with those in the sham-operated group (Figures 3(a) and 3(b)). Dectin-1 was involved in cerebral cell death after cerebral ischemia-reperfusion injury by mediating the apoptosis signal pathway.

### 3.3. Dectin-1-Mediated M1 Macrophage Polarization Is Involved in Cerebral Ischemia-Reperfusion Injury

The proinflammatory cytokines IL-6 and TNF-*α* were the symbolic cytokines of M1-polarized macrophages, while the anti-inflammatory cytokine IL-10 was known as the symbolic cytokine of M2-polarized macrophages [13]. The high level of M1-polarized macrophages was detected after cerebral ischemia-reperfusion injury, and the respective high level of dectin-1 may accelerate the polarization after cerebral ischemia-reperfusion. The results in the study showed the ratio of M2 macrophages increased during the process of ischemia-reperfusion injury. The ratio of the sham-operated group was 0.42%, while in the experimental group, it was 0.71%, 1.23%, and 1.82% after 6 hours, 12 hours, and 24 hours of ischemia-reperfusion injury, respectively (Figure 2).

## 4. Discussion

Currently, the study results showed that the dectin-1 expression levels were significantly increased in the brain tissue during the process of ischemia-reperfusion injury. At the same time, the expression of dectin-1 was significantly correlated with apoptosis-related proteins in cerebral ischemia/reperfusion models. Interestingly, the result of flow cytometry indicated that cerebral infarction was associated with the polarization of M1 macrophages and the high expression level of dectin-1. The levels of apoptosis-related proteins and the ratio of M1 macrophages supported the hypothesis that dectin-1 played an important role in cerebral infarction and apoptosis in the process of ischemia-reperfusion injury.

The high expression level of dectin-1 was observed on the bone marrow-derived macrophages and neutrophils during ischemic stroke. The protein deletion and expression tests showed that dectin-1 was significantly related to M1 macrophage polarization and neutrophil infiltration in myocardial infarcted tissue [10]. Laminarin, a dectin-1 antagonist, reduced the number of IBA1-positive cells and the expression of tumor necrosis factor and induced nitric oxide synthase on the third day after ischemic stroke, which reduced the volume of cerebral infarction and improved nerve function [14]. Upregulation of dectin-1 in myocardial cells after ischemia-reperfusion injury promoted cardiac remodeling by activating NF-*κ*B and NLRP3 [15]. Dectin-1 can cause demyelination and axonal injury in a macrophage-dependent way, and the dectin-1-null knockout animal model showed that the dieback of axons was also reduced after spinal cord injury [16]. Dectin-1 can mediate the inflammatory reaction in the injured area after ischemia-reperfusion injury, produce adverse effects, and compensate hyperplasia on the heart, brain, nerve, and other related tissues.

During myocardial ischemia-reperfusion injury, macrophages were shown to be the predominant cell type and displayed functional heterogeneity, with proinflammatory macrophages (M1 macrophages) infiltrating at the first time and then a high level of anti-inflammatory macrophages (M2 macrophages) at the second reaction, which was tended to the anti-inflammatory-related immune cells. M1 macrophages expressed TNF-*α*, iNOS, IL-1*β*, and IL-6, which induced a strong proinflammatory reaction and contributed to myocardial ischemia-reperfusion injury. In contrast, M2 macrophages expressed IL-10, ARG-1, and ARG-2 which depleted arginine stores and produced polyamine and proline (instead of nitric oxide), of which polyamine and proline were important for cell survival. Baicalein played a neuroprotective role in cerebral ischemia-reperfusion injury in vivo by regulating macrophage M1/M2 polarization [17].

In the rat model of pneumonia, high expression of dectin-1, which was a key receptor recognized and cleared by macrophages, induced the differentiation of macrophage M1 [18] in a NF-*κ*B-dependent pathway [19]. Dectin-1 derived from particulate *β*-glucan converted M2 bone marrow-derived macrophages into M1-like phenotype, which expresses proinflammatory cytokines such as TNF-*α*, iNOS, IL-1*β*, and IL-6 to inhibit the development of the tumor [20]. In addition, dectin-1 affects Syk, NF-*κ*B, and p38-associated pathways, which regulates neutrophil recruitment through the regulation of the expression and secretion of CXCL1 and G-CSF after ischemia-reperfusion injury [17].

In conclusion, dectin-1 is an important immune-cell-related receptor, of which the expression levels are increased in ischemia-reperfusion injury and correlated with the prognosis of ischemia-reperfusion injury. Dectin-1-mediated M1 macrophage polarization and brain cell apoptosis are involved in reperfusion injury. The research in this paper is still not thorough enough, and a part of the results is just correlation studies. In the future, we may obtain a more scientific research result through the construction of the cell model and the intervention of the protein expression.

## Data Availability

The analyzed datasets generated during this study are available from the corresponding author within 12 months of publication.
